# Δ^8(14)^-Ergostenol Glycoside Derivatives Inhibit the Expression of Inflammatory Mediators and Matrix Metalloproteinase

**DOI:** 10.3390/molecules26154547

**Published:** 2021-07-28

**Authors:** Hyejin Moon, Myoungsil Ko, Yujin Park, Jeonguk Kim, Dowon Yoon, Eunjoohwang Lee, Taehoon Lee, Hakwon Kim

**Affiliations:** 1 Global Center for Pharmaceutical Ingredient Materials, Department of Applied Chemistry, Kyung Hee University, Yongin 17104, Korea; hjm6719@naver.com (H.M.); kakddugy@gmail.com (M.K.); jungwoogi93@naver.com (J.K.); alslznls3152@naver.com (D.Y.); 2 Graduate School of East-West Medicinal Science, Kyung Hee University, Yongin 17104, Korea; pyj2386@hanmail.net (Y.P.); ehwang@khu.ac.kr (E.L.)

**Keywords:** ergostenol, ergostenol glycosides, arthritis, matrix metalloproteinase, collagen type II A1

## Abstract

Arthritis is a chronic inflammatory disease accompanied by pathological reactions such as swelling, redness, fever, and pain in various joint areas. The drugs currently available to treat arthritis are associated with diverse side-effects. Therefore, there is a need for safer and more effective treatments to alleviate the inflammation of arthritis with fewer side-effects. In this study, a new sterol, Δ^8(14)^-ergostenol, was discovered, and its glycosides were synthesized and found to be more efficient in terms of synthesis or anti-inflammatory activity than either spinasterol or 5,6-dihydroergosterol is. Among these synthetic glycosides, galactosyl ergostenol inhibited the expression of inflammatory mediators in TNF-α-stimulated FLS and TNF-α-induced MMPs and collagen type II A1 degradation in human chondrocytes. These results suggest the new galactosyl ergostenol as a treatment candidate for arthritis.

## 1. Introduction

Arthritis is a chronic inflammatory disease accompanied by pathological reactions such as swelling, redness, fever, and pain in various joint areas [1,2,3,4]. Joints are areas where two or more bones meet, and most of them are responsible for helping the body move [2]. In terms of anatomy, the structure of a joint is composed of cartilage, which protects the end of the bone, and a synovial membrane that secretes a clear sticky fluid around the joint [3]. Joint tissue inflammation is classified as either degenerative osteoarthritis (OA) or rheumatoid arthritis (RA) according to the loss of cartilage and autoimmune dysfunction of the synovial membrane [4].

The major cause of OA is age-related damage to chondrocytes. The primary pathological mechanism of OA is the reduced formation of the extracellular matrix of articular cartilage cells and the alteration of the extracellular matrix components of cartilage due to cellular aging [5]. Normal chondrocytes play an important role in cartilage homeostasis. Abnormal chondrocytes exposed to inflammatory cytokines and chemokines cause the excessive secretion of several types of matrix-degrading enzymes such as matrix metalloproteinases (MMPs). These MMPs break down collagen type II A1 (COL2A1), a major collagen component of the cartilage matrix. In particular, MMP-1, MMP-3, and MMP-13 play a major role in arthritis [6,7,8]. Rheumatoid arthritis is a chronic inflammatory condition that arises in the synovial membrane surrounding the joints. The major pathogenic mechanism of RA is the excessive secretion of inflammatory cytokines and chemokines due to immune abnormalities, leading to the gradual destruction of cartilage tissue with an increased production of matrix degradation enzymes such as MMPs [9].

The major constituents of the cartilage matrix are COL2A1 and proteoglycans. Degenerative osteoarthritis and rheumatoid arthritis are caused by the degradation of COL2A1, which is primarily mediated by MMP-1, MMP-3, and MMP-13. These MMPs are secreted mainly by human chondrocytes (HCs) and human fibroblast-like synoviocytes (FLSs) [4]. The over-expression of MMPs such as MMP-1, MMP-3, and MMP-13 leads to a vicious cycle of the activation of inflammatory cytokines and chemokines [10]. Regulating these inflammatory mediators can alleviate symptoms of arthritis. Currently, various drugs, including steroidal, nonsteroidal, and biological materials with analgesic or anti-inflammatory properties, are used to treat arthritis [11]. These drugs generally have a short duration of effect and low efficacy, and they can cause many side effects when used long term [12,13]. Thus, there is a need for safer and more effective arthritis treatments to alleviate joint inflammation and inhibit COL2A1 degradation through inhibition of MMPs.

**Figure 1:**
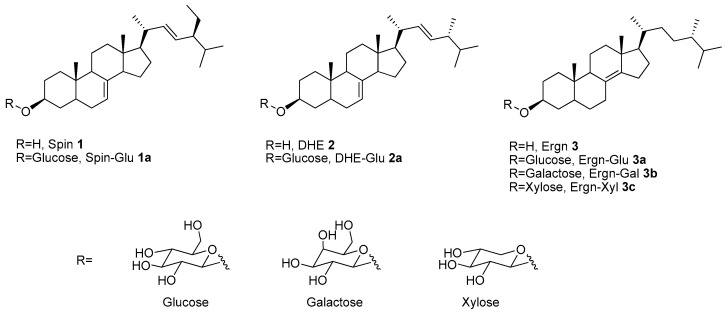
Structures of spinasterol (Spin, **1**), Spin-Glu (**1a**), 5,6-dihydroergosterol (DHE, **2**), DHE-Glu (**2a**), Δ^8(14)^-ergostenol (Ergn, **3**), and ergostenol glycoside (Ergn-Glu, **3a**; Ergn-Gal, **3b**; and Ergn-Xyl, **3c**).

Previously, we reported the potent anti-inflammatory activity of 5,6-dihydroergosterol glucose (DHE-Glu, **2a**), a synthetic compound derived from a natural product, spinasterol glucose (Spin-Glu, **1a**) [14]. In HaCaT cells, DHE-Glu (**2a**) produced strong anti-inflammatory activity through the inhibition of TNF-α/IFN-γ-induced expressions of CCL17 and CCL22, and also inhibited DNCB-induced contact dermatitis in an animal model of chronic inflammation [15]. As a follow-up to this study, we tried to find a new synthetic sterol that could be synthesized more efficiently and/or had a greater bioactivity than spinasterol (Spin, **1**) or 5,6-dihydroergosterol (DHE, **2**) did. As a result, Δ^8(14)^-ergostenol (Ergn, **3**), a sterol having a double bond at carbon position 8(14) of the steroid skeleton, was identified as a candidate compound. This sterol can be synthesized from ergosterol by selective hydrogenation followed by double bond migration [16]. We synthesized ergostenol glycosides containing a sugar, such as glucose, galactose, or xylose, by Schmidt glycosylation of Ergn (**3**) with a glycosyl imidate. Spin (**1**), Spin-Glu (**1a**), DHE (**2**), DHE-Glu (**2a**), Ergn (**3**), and ergostenol glycosides including Ergn-Glu (**3a**), Ergn-Gal (**3b**), and Ergn-Xyl (**3c**) are shown in Figure 1. In this study, newly synthesized ergostenol glycosides were tested for anti-inflammatory properties in FLSs and the suppression of MMPs expression in HCs. These results may suggest the possibility of being developed as a treatment for arthritis.

## 2. Materials and Methods

### 2.1. Chemicals and Instruments

The ^1^H NMR and ^13^C NMR spectra were recorded on a Jeol 300 MHz spectrometer and Jeol 75 MHz spectrometer, respectively. Chemical shifts (*δ*) were reported in parts-per-million (ppm), and coupling constants (*J*) were reported in Hertz (Hz). The following abbreviations were used to describe multiplicities: s = singlet, d = doublet, t = triplet, q = quartet, m = multiplet, and br s = broad singlet. Melting points (m.p.) were determined on a Barnstead Electrothermal 9100 instrument. The IR spectra were recorded on a Jasco FT/IR-4700 type instrument, while HR-FAB^+^-MS was recorded on a Jeol JMS-700 instrument. All chemicals were purchased from Sigma-Aldrich, Alfa Aesar, Acros Organics, and Tokyo Chemical Industry and used without further purification. Reaction progress was monitored by thin-layer chromatography (TLC, Merck kieselgel 60 F_254_), and column chromatography was performed using Merck silica gel 60 (particle size 0.040–0.063 mm). Extra pure-grade solvents for column chromatography were purchased through Samchun Chemicals and Duksan Chemicals. The 2,3,4,6-tetra-*O*-benzoyl-glucopyranosyl trichloroacetimidate (glucosyl imidate, **4a**), 2,3,4,6-tetra-*O*-benzoyl-galactopyranosyl trichloroacetimidate (galactosyl imidate, **4b**), and 2,3,4-tri-*O*-benzoyl-xylopyranosyl trichloroacetimidate (xylosyl imidate, **4c**) were prepared as described previously [17].

### 2.2. Synthesis of 5α-Ergost-8(14)-en-3β-ol (Ergn, ***3***)

Ergosterol (5 g, 12.61 mmol) in a mixture of methylene chloride (126 mL) and methanol (118 mL) was hydrogenated under H_2_ for 3 h in the presence of Pd/C (10% wt) catalyst (0.50 g, 0.4666 mmol). The mixture was filtered through celite, concentrated in vacuo, and purified by flash column chromatography (ethyl acetate/hexane) to produce Ergn **3** as a white solid.

Yield: 93 % m.p.: 131.2–132.4 °C (Lit.^ref^ m.p. 130–131 °C). ^1^H NMR (300 MHz, chloroform-*d*) *δ* (ppm): 0.69 (s, 3 H), 0.78 (d, *J* = 6.6 Hz, 6 H), 0.84 (s, 3H), 0.86 (d, *J* = 6.7 Hz, 3 H), 0.93 (d, *J* = 6.4 Hz, 3 H), 3.63 (br s, 1 H). ^13^C NMR (75 MHz, chloroform-*d*) *δ* (ppm): 12.9, 15.5, 17.7, 18.2, 19.3, 20.0, 20.6, 25.9, 27.1, 28.9, 29.7, 30.4, 31.6, 31.7, 33.6, 34.9, 36.6, 36.9, 37.3, 38.4, 39.2, 42.8, 44.4, 49.4, 56.8, 71.4, 126.5, 142.9. IR: OH peak at 3662 cm^−1^.

### 2.3. Synthesis of Ergostenol Glycosides (***3a***, ***3b***, and ***3c***) through Glycosylation and Hydrolysis

#### 2.3.1. Synthesis of 5α-Ergost-8(14)-en-3β-yl-β-d-Glucopyranoside (Ergn-Glu **3a**)

2,3,4,6-Tetrabenzoyl-β-glucopyranosyl ergostenol (**5a**): Glucosyl imidate (**4a**; 0.30 g, 0.3993 mmol), Ergn (**3**; 0.080 g, 0.1997 mmol), and a 4Å molecular sieve in anhydrous methylene chloride (3.99 mL) were stirred at 0 °C. After addition of trimethylsilyl trifluoromethanesulfonate (TMSOTf, 3.6 μL, 0.01997 mmol) at 0 °C, the mixture was stirred at room temperature for 1 h under an argon atmosphere. After neutralization by Et_3_N, the mixture was filtered to remove the molecular sieve, concentrated in vacuo, and purified by flash column chromatography (ethyl acetate/hexane) to produce a benzoyl-protected Ergn-Glu (**5a**) as a white solid. Yield: 80% m.p.: 185.6–187.3 °C. ^1^H NMR (300 MHz, chloroform-*d*) *δ* (ppm): 0.56 (s, 3 H), 0.76–0.82 (m, 9 H), 0.85 (d, *J* = 6.8 Hz, 3 H), 0.93 (d, *J* = 6.2 Hz, 3 H), 3.56–3.68 (m, 1 H), 4.13–4.20 (m, 1 H), 4.51 (dd, *J* = 11.9, 5.9 Hz, 1 H), 4.60 (dd, *J =* 12.1, 3.5 Hz, 1 H), 4.94 (d, *J* = 8.1 Hz, 1 H), 5.49 (dd, *J* = 9.7, 7.9 Hz, 1 H), 5.61 (t, *J* = 9.7 Hz, 1 H), 5.89 (t, *J* = 9.5 Hz, 1 H), 7.27–7.59 (m, 12 H), 7.82–7.84 (m, 2H), 7.89–7.92 (m, 2H), 7.94–7.97 (m, 2H), 8.00–8.03 (m, 2H).

Ergn-Glu (**3a**): Benzoyl-protected Ergn-Glu (**5a**; 3.31 g, 3.380 mmol) in a mixed solvent (CH_2_Cl_2_:MeOH = 67.6 mL:67.6 mL) was added to NaOMe (4.37 M in MeOH, 5.41 mL, 23.66 mmol). The mixture was stirred at room temperature for 5 h. After neutralization by Dowex Mac-3, it was filtered and concentrated in vacuo. The white residue was purified by flash column chromatography (CH_2_Cl_2_/MeOH) to recover Ergn-Glu (**3a**) as a white solid. Yield: 87% m.p.: 231.2–234.2 °C. ^1^H NMR (300 MHz, pyridine-*d_5_*) *δ* (ppm): 0.63 (s, 3 H), 0.82 (d, *J* = 3.9 Hz, 3 H), 0.84 (d, *J* = 3.9 Hz, 3 H), 0.87–0.91 (m, 6 H), 1.00 (d, *J* = 6.6 Hz, 3 H), 3.95–4.06 (m, 1 H), 4.06–4.14 (m, 2 H), 4.26–4.41 (m, 2 H), 4.47 (dd, *J* = 11.8, 5.4 Hz, 1 H), 4.67 (dd, *J* = 11.7, 2.0 Hz, 1 H), 5.11 (d, *J* = 7.7 Hz, 1 H). ^13^C NMR (75 MHz, pyridine-*d_5_*) *δ* (ppm): 12.9, 15.7, 17.8, 18.6, 19.6, 20.3, 20.7, 26.2, 27.4, 29.3, 30.0, 30.2, 30.8, 31.8, 33.9, 34.9, 35.2, 36.8, 37.2, 37.7, 39.5, 43.1, 44.2, 49.7, 57.1, 63.1, 72.0, 75.6, 77.1, 78.8, 78.8, 102.3, 127.1, 142.9 IR: OH peak at 3673 cm^−1^. HR-FAB^+^-MS m/z: 563.4316(M + 1) (Calcd for C_34_H_59_O_6_: 563.4312).

#### 2.3.2. Synthesis of 5α-Ergost-8(14)-en-3β-yl-β-d-Galactopyranoside (Ergn-Gal, **3b**)

2,3,4,6-Tetrabenzoyl-β-galactopyranosyl ergostenol (**5b**): Following the procedure described above for the preparation of **5a**, a benzoyl-protected Ergn-Gal (**5b**) was obtained as a white solid from galactosyl imidate (**4a**). Yield: 77% m.p.: 93.7–95.4 °C. ^1^H NMR (300 MHz, Chloroform-*d*) *δ* (ppm): 0.58 (s, 3 H), 0.77 (s, 3 H), 0.80 (d, *J* = 6.4 Hz, 6 H), 0.85 (d, *J* = 6.8 Hz, 3 H), 0.93 (d, *J* = 6.4 Hz, 3 H), 3.55–3.73 (m, 1 H), 4.27–4.36 (m, 1 H), 4.43 (dd, *J* = 11.1, 6.5 Hz, 1 H), 4.68 (dd, *J* = 11.2, 7.0 Hz, 1 H), 4.91 (d, *J* = 7.9 Hz, 1 H), 5.59 (dd, *J* = 10.4, 3.4 Hz, 1 H), 5.77 (dd, *J* = 10.3, 7.9 Hz, 1 H), 5.97–5.98 (m, 1 H), 7.34–7.53 (m, 10 H), 7.53–7.66 (m, 2 H), 7.74–7.82 (m, 2 H), 7.91–7.99 (m, 2 H), 7.99–8.06 (m, 2 H), 8.07–8.15 (m, 2 H).

Ergn-Gal (**3b**): Following the deprotection procedure described above for the preparation of **3a**, Ergn-Gal (**3b**) was obtained as a white solid from **4b**. Yield: 85%. m.p.: 241.8–245.2 °C. ^1^H NMR (300 MHz, pyridine-*d_5_*) *δ* (ppm): 0.62 (s, 3 H), 0.82 (d, *J* = 3.9 Hz, 3 H), 0.84 (d, *J* = 3.9 Hz, 3 H), 0.87–0.92 (m, 6 H), 1.00 (d, *J* = 6.6 Hz, 3 H), 3.95–4.10 (m, 1 H), 4.20 (t, *J* = 6.2 Hz, 1 H), 4.27 (dd, *J* = 9.2, 3.3 Hz, 1 H), 4.45–4.59 (m, 3 H), 4.64 (br. s., 1 H), 5.02 (d, *J* = 7.5 Hz, 1 H). ^13^C NMR (75 MHz, pyridine-*d_5_*) *δ* (ppm): 12.9, 15.7, 17.8, 18.6, 19.5, 20.3, 20.7, 26.2, 27.4, 29.3, 30.0, 30.2, 30.8, 31.8, 33.9, 35.0, 35.2, 36.8, 37.2, 37.7, 39.4, 43.1, 44.2, 49.7, 57.1, 62.8, 70.6, 72.9, 75.6, 76.9, 77.3, 102.9, 127.1, 142.8. IR: OH peak at 3673 cm^−1^. HR-FAB^+^-MS m/z: 563.4315(M + 1) (Calcd for C_34_H_59_O_6_: 563.4312).

#### 2.3.3. Synthesis of 5α-Ergost-8(14)-en-3β-yl-β-d-Xylopyranoside (Ergn-Xyl, **3c**)

2,3,4-Tribenzoyl-β-xylopyranosyl ergostenol (**5c**): Following the procedure described above for the preparation of **5a**, a benzoyl-protected Ergn-Xyl (**5c**) was obtained from xylosyl imidate (**4c**) as a white solid. Yield: 84% m.p.: 157.6–159.9 °C. ^1^H NMR (300 MHz, chloroform-*d*) *δ* (ppm): 0.62 (s, 3 H), 0.78 (d, *J* = 6.8 Hz, 6 H), 0.82 (s, 3 H), 0.85 (d, *J* = 7.0 Hz, 3 H), 0.93 (d, *J* = 6.4 Hz, 3 H), 3.69 (dd, *J* = 12.0, 7.1 Hz, 2 H), 4.45 (dd, *J* = 12.0, 4.3 Hz, 1 H), 4.97 (d, *J* = 5.5 Hz, 1 H), 5.25–5.37 (m, 2 H), 5.76 (t, *J* = 7.2 Hz, 1 H), 7.32–7.43 (m, 6 H), 7.47–7.58 (m, 3 H), 7.97–8.03 (m, 6 H).

Ergn-Xyl (**3c**): Following the deprotection procedure described above for the preparation of **3a**, Ergn-Gal (**3c**) was obtained as a white solid from **4c**. Yield: Quantitative m.p.: 204.2–207.2 °C. ^1^H NMR (300 MHz, pyridine-*d_5_*) *δ* (ppm): 0.64 (s, 3 H), 0.82 (d, *J* = 3.9 Hz, 3 H), 0.84 (d, *J* = 3.9 Hz, 3 H), 0.87–0.92 (m, 6 H), 1.00 (d, *J* = 6.4 Hz, 3 H), 3.83 (t, *J* = 10.3 Hz, 1 H), 3.89–4.01 (m, 1 H), 4.06 (t, *J* = 8.0 Hz, 1 H), 4.20–4.36 (m, 2 H), 4.45 (dd, *J* = 11.0, 4.8 Hz, 1 H), 4.95 (d, *J* = 7.5 Hz, 1 H). ^13^C NMR (75 MHz, pyridine-*d_5_*) *δ* (ppm): 13.0, 15.8, 18.0, 18.7, 19.7, 20.5, 20.9, 26.3, 27.6, 29.5, 30.1, 30.4, 31.0, 32.0, 34.0, 35.2, 35.3, 37.1, 37.4, 37.8, 39.6, 43.2, 44.5, 49.8, 57.3, 67.5, 71.6, 75.5, 77.6, 78.9, 103.4, 127.2, 143.0. IR: OH peak at 3673 cm^−1^ HR-FAB^+^-MS m/z: 533.4208(M + 1) (Calcd for C_33_H_57_O_5_: 533.4206).

### 2.4. Biological Evaluation of Materials

Chondrocyte growth media were purchased from Cell Signaling Technology (411–500, Beverly, MA, USA). Dulbecco’s modified Eagle’s medium (DMEM), fetal bovine serum (FBS), and antibiotics (penicillin/streptomycin) were purchased from Welgene (Kyungsan, Korea). Anti-human MMP-1 (SC 58377) and anti-MMP-13 (SC 515284), anti-INOS (SC 650), anti-COX-2 (SC 1746), anti-IL-1β (SC 7884), and anti-GAPDH (SC 166574) antibodies were purchased from Santa Cruz Biotechnology (Santa Cruz, CA, USA). Anti-MMP-3 antibody was purchased from R&D Systems (Minneapolis, MN, USA). The primers used in PCR analyses were obtained from Bioneer (Daejeon, Korea).

### 2.5. Cell Culture

Human chondrocytes (HCs) and fibroblast-like synoviocytes (FLSs) were purchased from the ATCC. HCs were cultured in chondrocyte growth medium. The FLSs were cultured in Dulbecco’s modified Eagle’s medium supplemented with 10% fetal bovine serum, 100 units/mL of penicillin, and 100 μg/mL of streptomycin at 37 °C in a humidified 95% and 5% (*v*/*v*) mixture of air and CO_2_.

### 2.6. Cell Viability Assay

Cell viability was measured using an Ez Cytox (Do Gen, Inc., Seoul, Korea) assay. Cells were seeded into 96-well plates, and the medium was replaced with 100 μL of fresh medium containing ergostenol glycosides. The cells were incubated for 24 h at 37 °C in 5% CO₂. Next, 10 μL of Ez Cytox reagent was added to each well, and the plate was incubated at 37 °C for 1 h. The absorbance was measured at 570 nm using a spectrophotometer (Thermo Fisher Scientific, Waltham, MA, USA).

### 2.7. Reverse Transcription-Polymerase Chain Reaction (PCR)

RNA was harvested using RNA Bee (Friendswood, TX, USA) according to the manufacturer’s instructions. Total RNA (5 μg) was used for cDNA synthesis. The PCR was performed using a PTC-100 Peltier thermal cycler (MJ Research, Hercules, CA, USA) in accordance with the general protocol. The cycle profile used for PCR was as follows: initial denaturation step at 95 °C for 10 min, followed by 40 cycles at 95 °C for 20 s, 60 °C for 15 s, and 72 °C for 20 s. GAPDH was used as a loading control. PCR products were electrophoretically separated on 1% agarose gel containing 0.5 μg/mL of ethidium bromide and were visualized with an ultraviolet light transilluminator. The primer sequences used are shown in Table 1.

### 2.8. Western Blotting

The cell extracts from each cell were prepared in RIPA buffer (Pierce Biotechnology, Rockford, IL, USA). Protein concentrations were measured using the Bradford protein assay, and the proteins were fractionated by electrophoresis in 10% gel and transferred to a nitrocellulose membrane. To prevent nonspecific binding, the membrane was soaked in buffer containing 5% dry skim milk for 1 h. Membranes were incubated with anti-MMP-1, anti-MMP-3, anti-MMP-13, anti-iNOS, anti-COX-2, anti-IL-1β, and anti-GAPDH. Antibodies were incubated overnight at 4 °C. Membranes were washed and incubated with secondary antibody (goat-anti-mouse and goat-anti-rabbit IgG; 7074P2, 7074P6; Cell signaling biotechnology, Danvers, MA, USA) for 1 h at room temperature. Membranes were visualized with standard ECL reagent and measured using a chemiluminescence detection system (Thermo Fisher Scientific, Waltham, MA, USA).

### 2.9. Enzyme-Linked Immunosorbent Assay

Human chondrocytes (HCs) and fibroblast-like synoviocytes (FLSs) were pre-incubated in 6-well cell culture plates and treated with TNF-α (10 ng/mL) in the presence or absence of Ergn-Gal (**3b**) for 18 h. We collected the cell culture supernatants by centrifugation at 1500× *g* for 10 min to obtain cell-free supernatants and analyzed them using enzyme-linked immunosorbent assay (ELISA) kits (R&D Systems, Minneapolis, MN, USA) according to the manufacturer’s instructions.

### 2.10. Statistical Analysis

Unless otherwise stated, all experiments were performed with triplicate samples and repeated at least three times. Data are presented as means ± S.D. and statistical comparisons between groups were performed using one-way ANOVA followed by a Student’s *t*-test.

## 3. Results

### 3.1. Synthesis of *Δ*^8(14)^-Ergostenol Glycosides (Ergn-Gly, ***3a***, ***3b***, and ***3c***)

**Scheme 1:**
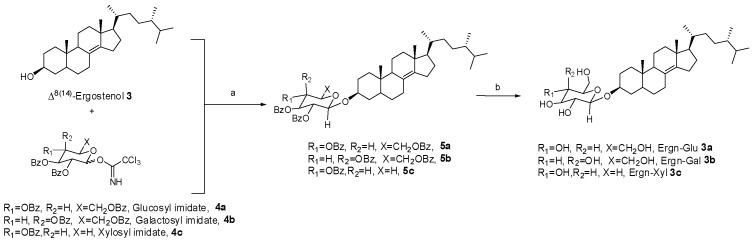
Synthesis of ergostenol glycosides: Ergn-Glu (**3a**), Ergn-Gal (**3b**), and Ergn-Xly (**3c**); reagents and conditions: (**a**) TMSOTf (0.10 eq), CH_2_Cl_2_, 0 °C to room temperature for 1 h, and (**b**) NaOMe (4.37 M in MeOH, 7.0 eq), CH_2_Cl_2_, MeOH at room temperature for 5 h.

In our previous work, DHE (**2**), prepared using the DIBAL-H (diisobutylaluminium hydride) reduction of ergosterol, was used as a substitute for natural spinasterol (Spin, **1**) [18]. During the course of this project, we tried to synthesize a sterol without double bonds in the steroid backbone to see how the double bonds affected the physiological activity of Spin (**1**) and DHE (**2**). Therefore, to prepare sterols with reduced double bonds in the steroid backbone, as has occurred in stigmastanol, Pd-catalyzed hydrogenation of DHE (**2**) was performed. However, while the double bond in the side chain was broken, another double bond in the steroid backbone moved from position C-7 to position C-8(14). The presence of double bonds was not confirmed in the ^1^H NMR spectrum, but the presence and migration of a double bond were confirmed in the ^13^C NMR spectrum (*δ* 126.5, 142.9). Finally, we confirmed that the structure of the Pd-catalyzed hydrogenation product of DHE (**2**) was Ergn (**3**) from our spectral data and the literature [19]. It was assumed that the mechanism by which DHE (**2**) was converted to Ergn (**3**) involved isomerization of the double bond at position C-7 instead of hydrogenation. Therefore, as DHE (**2**) was produced by reducing the C-5 double bond of ergosterol by a selective hydrogenation reaction, it was assumed that if direct Pd-catalyzed hydrogenation of ergosterol was carried out, the hydrogenation reaction and the subsequent isomerization reaction would occur successively, resulting in Ergn (**3**). Compared to Spin (**1**) or DHE (**2**), Ergn (**3**) has been shown to support the good synthesis and bioactivity of sterol and can be efficiently synthesized from commercially available ergosterol in one step [20]. We decided to use Ergn (**3**) as a novel sterol for the development of new anti-inflammatory drugs. Glycosides were prepared from Ergn (**3**) using the Schmidt glycosylation method, as previously described [21]. The Schmitt glycosylation reaction for Ergn (**3**) was carried out using the corresponding glycosyl imidate as the glycosyl donor in the presence of TMSOTf as Lewis acid catalyst [22]. In this reaction, the β-anomer of sterol glycosides is the major product of neighboring group participation [23]. The TMSOTf-catalyzed glycosylation of Ergn (**3**) by glycosyl imidate (glucosyl imidate **4a**, galactosyl imidate **4b**, and xylosyl imidate **4c**) yielded β-linked glycosyl ergostenol intermediates (**5a**, **5b**, and **5c**). The subsequent deprotection yielded Ergn-Glu (**3a**), Ergn-Gal (**3b**), and Ergn-Xly (**3c**), respectively (Scheme 1).

### 3.2. Comparison of the Structures of DHE (***2***) and Ergn (***3***) and Biological Activities of their Glycosides

**Figure 2:**
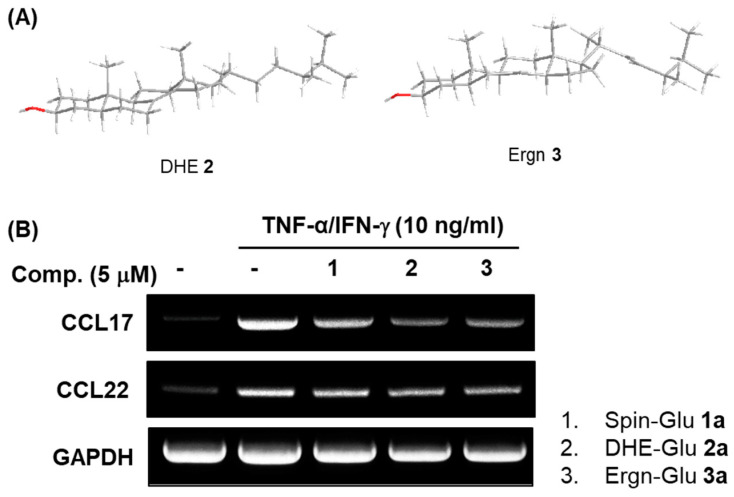
(**A**) Optimized structures of DHE (**2**, left) and Ergn (**3**, right) by DFT calculation (Gaussian B3LYP, 6331[d,p]). Grey (carbon), white (hydrogen), and red (oxygen) bonds. (**B**) RT-PCR assay was performed to compare the effects of Spin-Glu (**1a**), DHE-Glu (**2a**), and Ergn-Glu (**3a**) on CCL17 and CCL22 mRNA expressions.

Next, we verified the structural stability of DHE (**2**) and Ergn (**3**) according to density functional theory (DFT). The optimized structures of Ergn (**3**) and DHE (**2**) obtained with the Gaussian 09 program [B3LYP, 6-311+G(d, p)] are shown in Figure 2A and found to be similar to each other [24].

Therefore, the two compounds with similar backbone structures were expected to have similar biological activities. Figure 2B presents the comparisons of the inhibition of the TNF-α-induced CCL17 and CCL22 mRNA expression in human keratinocytes by synthetic compounds with different backbones, including Spin-Glu (**1a**), DHE-Glu (**2a**), and Ergn-Glu (**3a**). As a result, as we have reported, Spin-Glu (**1a**) and DHE-Glu (**2a**) inhibited the expressions of CCL17 and CCL22 mRNA in HaCaT cells induced by TNF-α. Furthermore, we confirmed that the newly synthesized Ergn-Glu (**3a**) produced similar inhibitions of CCL17 and CCL22 mRNA expressions in TNF-α-induced HaCaT cells.

### 3.3. Effect of Ergostenol Glycosides on the Viability of Human Chondrocytes (HCs) and Human Fibroblast-Like Synoviocytes (FLSs)

**Figure 3:**
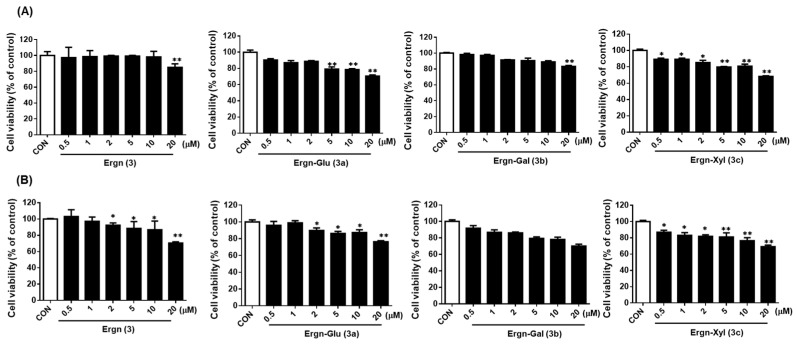
Effects of newly synthesized ergostenol glucosides on cell viability in FLSs and HCs. (**A**) Human fibroblat like synoviocytes (FLSs) (**B**) Human chondrocytes (HCs). Cell viability assays were performed using a concentration up to 20 μM ergostenol glycoside. Cell survival was confirmed using the MTT assay, and the results were measured based on OD value. The viability tests were performed in triplicate and repeated at least three times. * *p* < 0.05 and ** *p* < 0.01 compared with the negative control.

We used the MTT assay to verify the effect of ergostenol glycosides on cell viability in HCs and FLSs. As Ergn (**3**) has a structural similarity to DHE (**2**), the treatment concentrations of their glycosides were determined to be 0.5 to 20 μM. The viability of cell cultures incubated with only the medium without growth supplement was expressed as 100%, and cell viability was measured after ergostenol glycosides were applied under the same conditions. The ergostenol glycosides affected cell survival slightly in a concentration-dependent manner in both cell types, as shown in Figure 3, but approximately 80% cell survival was maintained even at the highest treatment concentration of 20 μM.

### 3.4. Ergostenol Glycosides Inhibited TNF-α-Induced Expression of the Pro-Inflammatory Cytokines in Human Fibroblast-Like Synoviocytes (FLSs)

**Figure 4:**
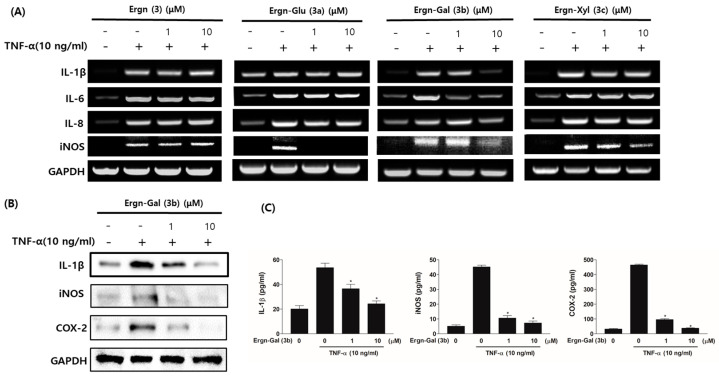
Effects of Ergn-Gal (**3b**) on mRNA and protein expression of pro-inflammatory cytokine and immune-modulating factors in TNF-α-induced FLSs. (**A**) RT-PCR assay was performed to compare the mRNA expression of pro-inflammatory cytokine and immune-modulating factors. (**B**) Western blotting was performed to compare IL-1β, iNOS, and COX-2 expressions. (**C**) TNF-α, iNOS, and COX-2 amounts of protein in cell culture supernatant detected using ELISA. * *p* < 0.05 compared with the only TNF-α-treated cell supernatant.

Both IL-1β and IL-8 have been identified as inflammatory mediators associated with OA and RA, and iNOS is considered a signaling mediator. In addition, COX-2 is a pro-inflammatory enzyme in arthritis [25,26,27,28]. Thus, we examined the effects of ergostenol glycosides on pro-inflammatory mediators in cell culture models of arthritis. Each cell type was stimulated with 10 ng/mL of TNF-α, and the cell cultures were incubated with ergostenol glycosides at various concentrations. The expression of pro-inflammatory cytokine (IL-6, IL-1β, IL-8, and iNOS) mRNA was measured in the TNF-α-induced FLSs to evaluate the effects of the ergostenol glycosides. At concentrations of 1 or 10 μM, Ergn-Gal (**3b**) strongly inhibited the expression of TNF-α-induced pro-inflammatory cytokine mRNA in a concentration-dependent manner. However, we confirmed that the iNOS mRNA expression was strongly inhibited by all tested compounds except Ergn (**3**), unlike the effects of other pro-inflammatory cytokines on mRNA expression levels (Figure 4A). Next, the effects of Ergn-Gal (**3b**) on pro-inflammatory cytokine protein expression were investigated in the TNF-α-stimulated FLSs. We assessed the effects of Ergn-Gal (**3b**) on the expression of pro-inflammatory cytokine proteins. IL-1β and iNOS were selected to verify the mRNA results, and COX-2 was selected as an important biomarker of arthritis. Similar to the mRNA results, Ergn-Gal (**3b**) strongly inhibited TNF-α-induced IL-1β and iNOS protein expressions, and the COX-2 protein expression was inhibited in a concentration-dependent manner (Figure 4B).

### 3.5. Matrix-Protective Effects of Ergostenol Glycosides in TNF-α-Induced HCs

**Figure 5:**
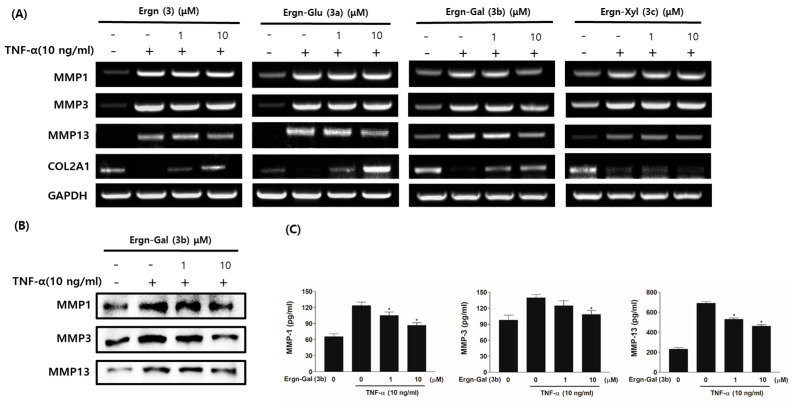
Effects of Ergn-Gal (3b) on MMPs and COL2A1 mRNA and protein expression in TNF-α-induced HCs. (**A**) RT-PCR assay was performed to compare MMPs and COL2A1 mRNA expressions. (**B**) Western blotting was performed to compare the expression of the MMPs. (**C**) MMP-1, MMP-3, and MMP13 amounts of protein in cell culture supernatant detected using ELISA. * *p* < 0.05 compared with the only TNF-α-treated cell supernatant.

The release of TNF-α induces an inflammatory response, and TNF-stimulated cells contribute to cartilage damage and synovitis. Thus, each cell was incubated with TNF-α for 24 h and then with ergostenol glycosides at various concentrations. Subsequent RT-PCR analyses indicated that Ergn-Gal (**3b**) inhibited the expression of TNF-α-induced MMP-1, MMP-3, and MMP-13 mRNA in HCs, while Ergn (**3**), Ergn-Glu (**3a**), and Ergn-Gal (**3b**) increased the expression of COL2A1 mRNA (Figure 5A). Western blot analyses indicated that the expression of TNF-α-induced MMPs in HCs was inhibited by Ergn-Gal (**3b**; Figure 5B), consistent with the RT-PCR results. Overall, these data suggest that Ergn-Gal (**3b**) exerts matrix-protective effects in cell culture models of arthritis.

## 4. Discussion

The most common types of arthritis are osteoarthritis (OA) and rheumatoid arthritis (RA). The major pathologic feature of OA is cartilage destruction, while RA is characterized by synovitis. In OA, chondrocytes are exposed to inflammatory cytokines and are activated. These activated chondrocytes produce MMPs, which induce cartilage damage and joint destruction [29,30]. The stimulation of FLSs in RA induces inflammatory mediators and MMPs, indicating the importance of FLSs in the propagation of inflammation and joint damage [31]. The drugs currently used to treat arthritis include nonsteroidal analgesic and anti-inflammatory drugs, such as azathioprine and prednisolone. However, these drugs are associated with diverse side-effects, including gastrointestinal symptoms, leucopenia, liver toxicity, pancreatitis, and spinal bone loss, depending on the dose and duration of drug use [12,13]. Therefore, there is a need for safer and more effective treatments that can alleviate the inflammation of arthritis with fewer side-effects.

In this study, we found that the sterol Ergn (**3**), having a double bond at carbon position 8(14) of the steroid skeleton, could be used for the synthesis of novel sterol glycosides. As shown in Figure 2A, the energy calculations (Gaussian B3LYP, 6-311 [d, p]) for the predicted Ergn (**3**) and DHE (**2**) structures were very similar, and the stability of Ergn (**3**) was 3.43 kcal/mol greater than that for DHE (**2**). These findings suggest that the energy difference between the two isomers underlies the differences in biological activity and the difference in the ease of synthesis, suggesting Ergn (**3**) as a suitable substitute for DHE (**2**).

Furthermore, a highly efficient method for Ergn (**3**) synthesis was developed, and ergostenol glycosides (**3a**, **3b**, and **3c**) containing a sugar, such as glucose, galactose, or xylose, were prepared via Schmidt glycosylation with a glycosyl imidate (**4a**, **4b**, or **4c**). We confirmed that ergostenol glycosides (**3a**, **3b**, and **3c**) have anti-inflammatory and matrix-protective effects in cell culture models of arthritis. Previous studies have shown that collagen type Ⅱ is a major component of the cartilage matrix, and MMPs are the major enzymes responsible for the degradation of cartilage matrix components [32]. As shown in Figure 4 and Figure 5, among ergostenol glycosides, Ergn-Gal (**3b**) was the most potent inhibitor of TNF-α-induced MMPs and collagen type II degradation in HCs. These results demonstrate Ergn-Gal (**3b**) as a promising matrix-protective agent. In addition, previous reports have shown that the prime etiologic agent of RA is TNF-α. In RA, FLSs produce or respond to inflammatory mediators, including IL-1β, IL-6, IL-8, iNOS, and COX-2. Therefore, RA FLSs have been reported to play a direct role in escalating inflammation [25,26,27,28].

We found that the assessed inflammatory mediators were inhibited by Ergn-Gal (**3b**) in TNF-α-stimulated FLSs. Curcumin, resveratrol, guggulsterone, withanolide, 6-shogaol, and boswellic acid are natural active ingredients known to alleviate arthritis through anti-inflammatory effects or inhibition of MMPs expression [33,34,35,36,37,38]. In this study, Ergn-Gal (**3b**) prevented collagen type Ⅱ degradation, inhibited inflammatory cytokines, and down-regulated MMPs expression, indicating its applicability as a superior candidate for treatment of arthritis. Pro-inflammatory cytokines, pro-inflammatory enzymes, and MMPs are regulated by the NF-κB signaling pathway, and the inhibition of NF-κB activation has been reported to be a potential target in the treatment of arthritis [39]. Therefore, further studies are needed to determine the function of Ergn-Gal (**3b**) as an anti-inflammatory agent that inhibits NF-κB in in vitro and in vivo experiments.

## 5. Conclusions

In conclusion, of the newly synthesized ergostenol glycosides in this study, Ergn-Gal (**3b**) exhibited the most potent anti-inflammatory activity. These results suggest that Ergn-Gal (**3b**) should be included in the list of new compounds in development for treating arthritis and other inflammatory diseases.

## Figures and Tables

**Table 1:** Specific primer sequences.

Gene		Sequence (5′-3′)	Size (bp)
MMP-1	sense antisense	CTGTTCAGGACAGAATGTG TTGGACTCACACCATGTGTT	395
MMP-3	sense antisense	TGCGTGGCAGTTTGCTCAGC GAATGTGAGTGGAGTCACCT	573
MMP-13	sense antisense	GGCTCCGAGAAATGCAGTCT ATCAAATGGGTAGAAGTCGC	273
COL2A1	sense antisense	AATCCAGCAAACGTTCCCAA CGGTACTCGATAACAGTCAA	380
IL-1β	sense antisense	ATGTACCAGTTGGGGAACTG GGATATGGAGCAACAAGTGG	264
IL-6	sense antisense	TGACAAACAAATTCGGTACATCC ATCTGAGGTGCCCATGCTAC	497
gIL-8	sense antisense	ATGACTTCCAAGCTGGCCGTGGCT TCTCAGCCCTCTTCAAAAACTTCTC	292
INOS	sense antisense	GGCCTCTCAGCTCACCCCGA CCAGGCGCACTGTCTGGTGG	496
GAPDH	sense antisense	AGGTCGGAGTCAACGGATTTGG ACAGTCTTCTGGGTGGCAGT	226

## Data Availability

The data presented in this study are available in this article or Supplementary Materials.

## Supplementary Materials

The following are available online: NMR of all compounds.

## Sample Availability

Samples of the all compounds are available from the authors.
