# Supplementary material for: Δ8(14)-Ergostenol Glycoside Derivatives Inhibit the Expression of Inflammatory Mediators and Matrix Metalloproteinase
Source: Molecules. 2021 Jul 28;26(15):4547. doi: 10.3390/molecules26154547 (PMC8347845; doi:10.3390/molecules26154547)
Supplement: Supplementary file 1 [file molecules-26-04547-s001.zip › molecules-1302901-supplementary.pdf]

Supporting Information

1. The cartesian coordinates for DHE (**2**) and Ergn (**3**) as below.

DHE (**2**)

Row	Symbol	X	Y	Z
1	C	4.1755510	2.0528860	0.4500790
2	C	4.7748450	0.8872690	-0.3442210
3	C	4.1188370	-0.4663250	0.0633320
4	C	2.5995310	-0.3836400	-0.3091720
5	C	1.9581070	0.9568650	0.0548630
6	C	2.6727330	2.0259880	0.4186510
7	H	4.5027460	1.0496920	-1.3975550
8	H	4.5483460	2.0441360	1.4839540
9	H	4.5275640	3.0006460	0.0247290
10	H	2.5482430	-0.4473030	-1.4083630
11	H	2.1603370	2.9551800	0.6510830
12	C	4.7888720	-1.5971300	-0.7598400
13	C	6.3092030	0.8726530	-0.2678580
14	C	6.9180030	-0.2642770	-1.0787320
15	C	6.3213090	-1.6087740	-0.6594130
16	H	4.3967220	-2.5699930	-0.4490460
17	H	6.7257470	-2.4085060	-1.2925050
18	H	4.5070640	-1.4732580	-1.8137600
19	H	6.6474770	-1.8239030	0.3635000
20	H	6.6503190	0.7721580	0.7683740
21	H	6.7078220	1.8249740	-0.6322830
22	H	6.6926500	-0.0962920	-2.1449160
23	C	1.7663500	-1.5774060	0.2262300
24	H	2.1313720	-2.5022330	-0.2302220

25	H	1.9286540	-1.6859770	1.3006420
26	C	0.4688130	0.9974870	-0.1756940
27	H	0.3410120	0.8047920	-1.2541540
28	C	0.2513770	-1.4676630	-0.0279610
29	H	0.0524530	-1.5178160	-1.1061470
30	H	-0.2375400	-2.3369230	0.4181760
31	C	-0.3278330	-0.1486520	0.5159820
32	C	-1.7782630	0.2139650	0.0554490
33	H	-1.8518160	-0.0759050	-1.0011940
34	C	-0.3337320	2.2645050	0.1321420
35	H	-0.0603980	2.6687050	1.1105900
36	H	-0.1537640	3.0550200	-0.6000620
37	C	-1.8066690	1.7766710	0.1302810
38	H	-2.3667260	2.1932090	-0.7091260
39	H	-2.3203040	2.1071120	1.0380150
40	C	4.3085580	-0.7325200	1.5739110
41	H	4.0599670	-1.7656620	1.8265800
42	H	5.3408770	-0.5683300	1.8871980
43	H	3.6748110	-0.0809620	2.1793540
44	C	-0.2011250	-0.0763350	2.0512100
45	H	-0.6966330	0.8073570	2.4597310
46	H	-0.6542270	-0.9520530	2.5239810
47	H	0.8421880	-0.0305660	2.3675990
48	C	-2.9936430	-0.4390800	0.7753220
49	H	-2.9474490	-0.1245120	1.8278680
50	C	-2.9744690	-1.9725160	0.7499250
51	H	-2.8933400	-2.3595430	-0.2702840
52	H	-2.1366820	-2.3698620	1.3231220
53	H	-3.8904010	-2.3757230	1.1894810
54	C	-6.5087650	0.0632640	-1.0268230
55	H	-6.4792250	-0.1597110	-2.1043330
56	C	-7.7479810	-0.7165960	-0.4836920

57	H	-7.5065720	-1.7808500	-0.6060750
58	C	-8.0064370	-0.4760310	1.0101440
59	H	-7.1088780	-0.6572550	1.6064370
60	H	-8.3348520	0.5502560	1.2003760
61	H	-8.7937650	-1.1421360	1.3749610
62	C	-9.0133510	-0.4471610	-1.3107460
63	H	-8.8402380	-0.6273370	-2.3762300
64	H	-9.8297990	-1.1011690	-0.9907790
65	H	-9.3607620	0.5839720	-1.1969120
66	C	-6.6284640	1.5857550	-0.8877730
67	H	-5.7813570	2.0845850	-1.3653090
68	H	-7.5375090	1.9542670	-1.3677730
69	H	-6.6517660	1.9023890	0.1581360
70	O	8.3323100	-0.2168030	-0.8751550
71	H	8.7286920	-0.9303050	-1.3851070
72	C	-4.2807840	0.1409700	0.2171390
73	H	-4.4050510	1.2070010	0.3839070
74	C	-5.2329810	-0.5118400	-0.4499800
75	H	-5.1200450	-1.5809410	-0.6209500

ERGN (**3**)

Row	Symbol	X	Y	Z
1	C	-4.1605480	-2.1836850	0.0474430
2	C	-4.9111340	-0.9209450	-0.3852290
3	C	-4.1616360	0.3764870	0.0513120
4	C	-2.7389700	0.3483390	-0.6183520
5	C	-1.9700430	-0.9194730	-0.2673190

6	H	-4.9061810	-0.9111350	-1.4866390
7	H	-4.1311450	-2.2469750	1.1406420
8	H	-4.7036600	-3.0703370	-0.2978770
9	H	-2.9381670	0.2829920	-1.7012320
10	C	-4.9668920	1.5875650	-0.4818530
11	C	-6.3863150	-0.9395950	0.0455490
12	C	-7.1432750	0.2801250	-0.4665470
13	C	-6.4419930	1.5747950	-0.0513380
14	H	-4.5026180	2.5216140	-0.1535800
15	H	-6.9621370	2.4349840	-0.4913810
16	H	-4.9158080	1.5853330	-1.5790620
17	H	-6.5314600	1.6774170	1.0352080
18	H	-6.4820110	-0.9709250	1.1360460
19	H	-6.8720780	-1.8446000	-0.3330490
20	H	-7.1741620	0.2346350	-1.5677600
21	C	-1.9182020	1.6309640	-0.3866380
22	H	-2.2922630	2.4258830	-1.0380720
23	H	-2.0568850	1.9901750	0.6361610
24	C	-0.4195420	1.4378030	-0.6429580
25	H	-0.2625880	1.1526790	-1.6903370
26	H	0.0893870	2.3930040	-0.4975950
27	C	0.1796780	0.3397920	0.2578120
28	C	1.5845470	-0.1959880	-0.2113690
29	H	1.5253070	-0.2804900	-1.3066140
30	C	0.1735050	-2.1486360	0.3401270
31	H	-0.0853270	-2.6820930	1.2612220
32	H	0.0357030	-2.8630250	-0.4768820
33	C	1.6253890	-1.6309420	0.3720490
34	H	2.2920670	-2.2880450	-0.1875450
35	H	2.0064440	-1.5989850	1.3983160
36	C	-4.0303690	0.4660120	1.5882980
37	H	-3.7365640	1.4718850	1.8983040

38	H	-4.9702600	0.2412870	2.0945320
39	H	-3.2770270	-0.2271380	1.9674520
40	C	0.2314410	0.7958900	1.7362150
41	H	0.6412440	0.0156710	2.3824650
42	H	0.8512790	1.6886930	1.8594170
43	H	-0.7674070	1.0266200	2.1097880
44	C	2.8588720	0.6244210	0.1214640
45	H	2.9619650	0.6441660	1.2147850
46	C	2.7968160	2.0775730	-0.3762640
47	H	2.5741510	2.1151220	-1.4477490
48	H	2.0354700	2.6596870	0.1429870
49	H	3.7482890	2.5901620	-0.2193960
50	C	6.7047650	-0.2404660	-0.4807660
51	H	6.5881200	-0.2177870	-1.5737050
52	C	7.9960870	0.5725930	-0.1794490
53	H	7.7738130	1.6057840	-0.4784360
54	C	8.3812240	0.6024690	1.3081100
55	H	7.5690950	0.9775560	1.9356830
56	H	8.6543680	-0.3926900	1.6714730
57	H	9.2453110	1.2546960	1.4650350
58	C	9.1867920	0.1099110	-1.0330580
59	H	8.9295870	0.0866110	-2.0964630
60	H	10.0367300	0.7877800	-0.9106860
61	H	9.5272740	-0.8903030	-0.7491560
62	C	6.8055160	-1.7164480	-0.0613470
63	H	5.9473290	-2.2891850	-0.4196790
64	H	7.6998920	-2.1937460	-0.4689850
65	H	6.8369060	-1.8210860	1.0274010
66	O	-8.4733140	0.2006330	0.0523180
67	H	-8.9620910	0.9651000	-0.2685510
68	C	4.1202490	-0.0769270	-0.4429090
69	H	4.1185440	0.0263420	-1.5369820

70	H	4.0567260	-1.1465950	-0.2383510
71	C	5.4542030	0.4431430	0.1148580
72	H	5.4520670	0.3181140	1.2050440
73	H	5.5378100	1.5192700	-0.0692310
74	C	-0.6889680	-0.9146420	0.1255600
75	C	-2.7320330	-2.2044560	-0.5199310
76	H	-2.8039000	-2.3513020	-1.6088050
77	H	-2.1896090	-3.0659580	-0.1275390

2. ^{1}H NMR and ^{13}C NMR spectra

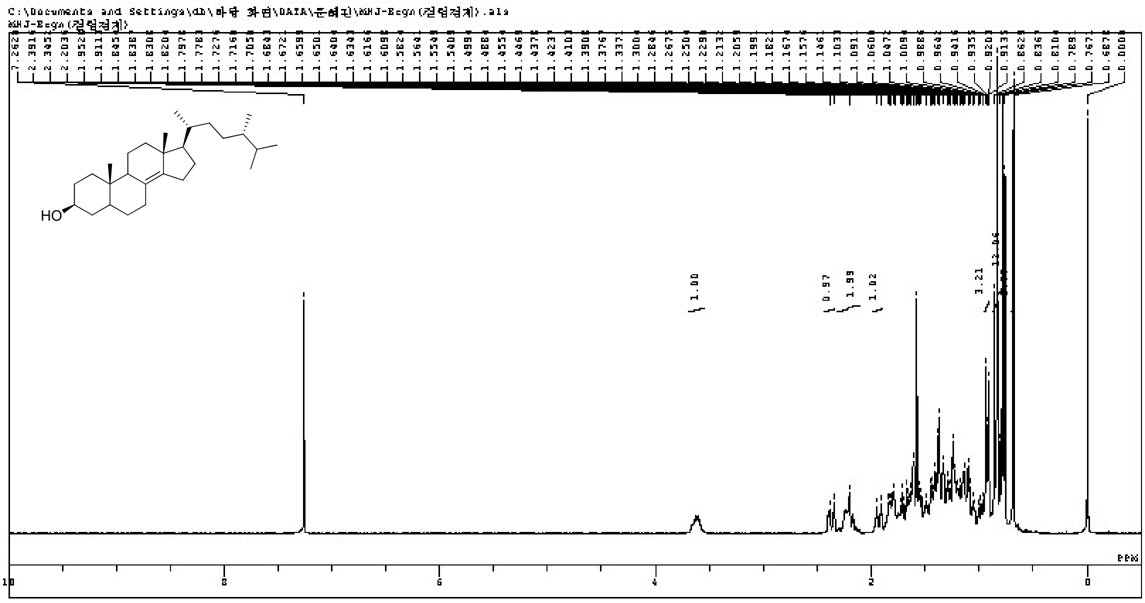

Figure S1. ^{1}H NMR spectrum of compound **3**

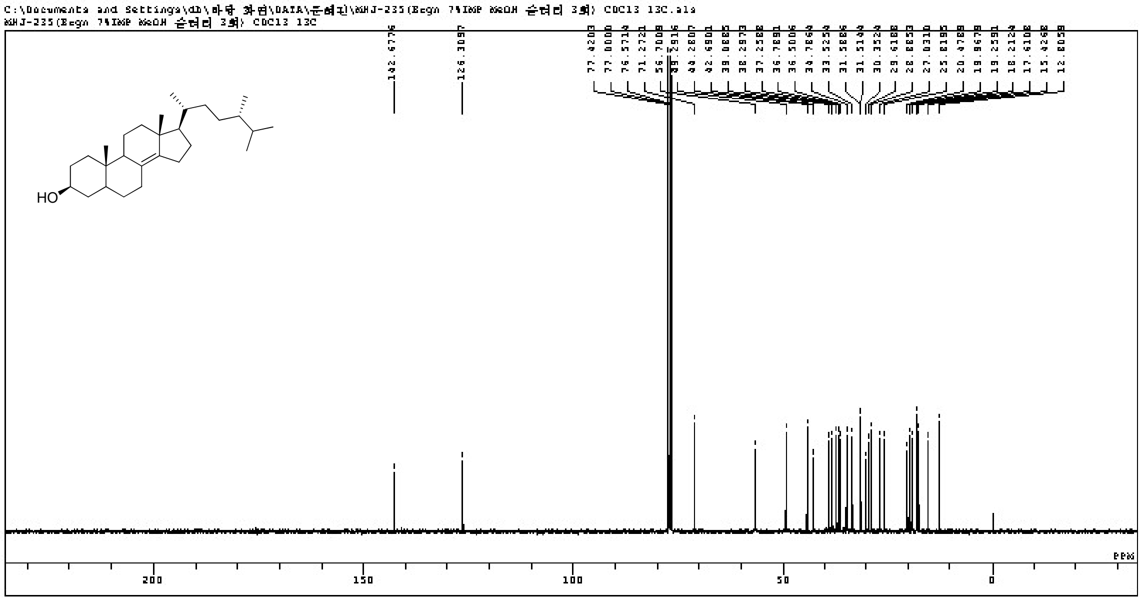

Figure S2. ^{13}C NMR spectrum of compound **3**

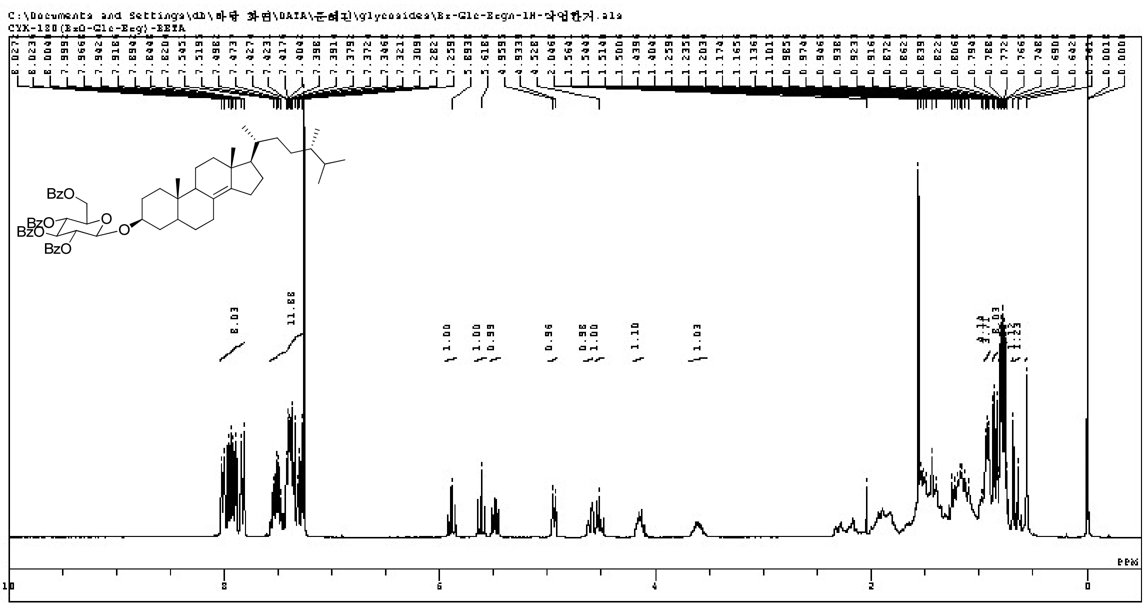

Figure S3. ^{1}H NMR spectrum of compound **5a**

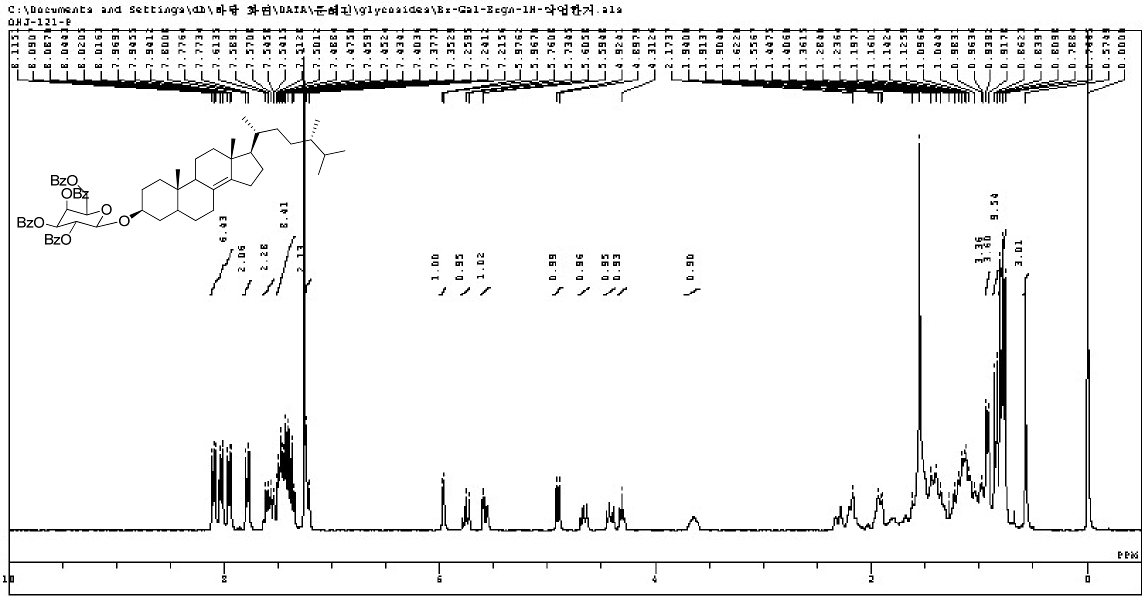

Figure S4. ^{1}H NMR spectrum of compound **5b**

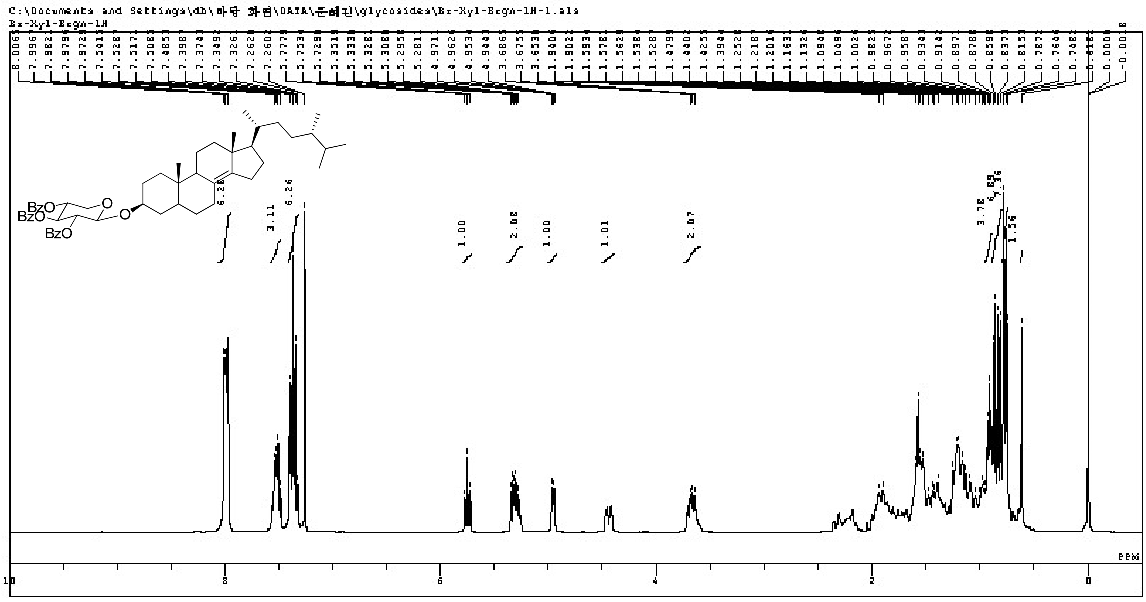

Figure S5. ^{1}H NMR spectrum of compound **5c**

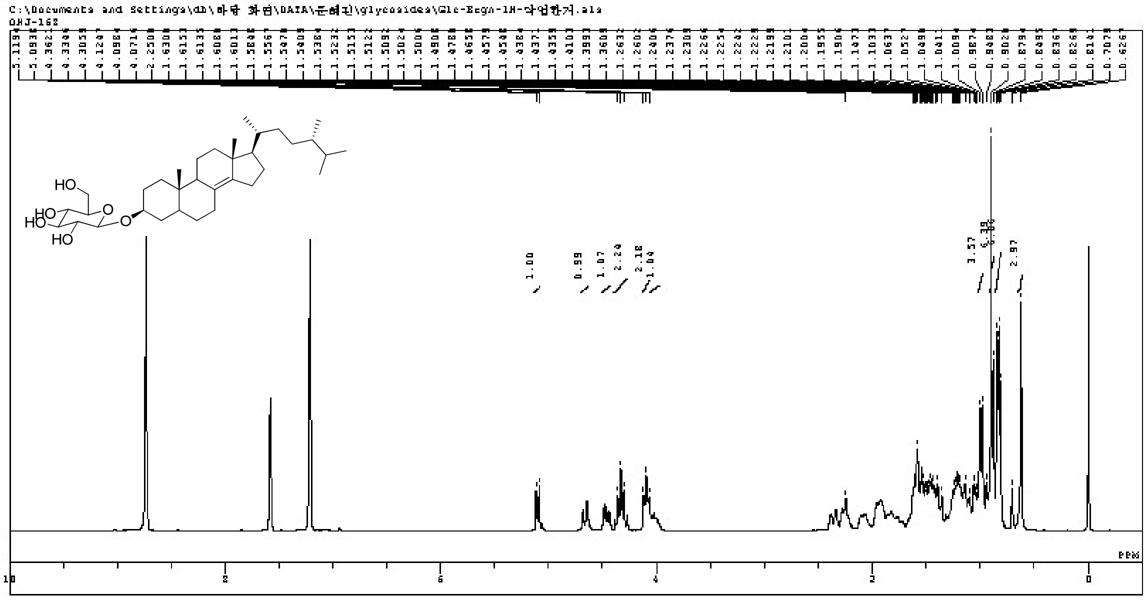

Figure S6. ^{1}H NMR spectrum of compound **3a**

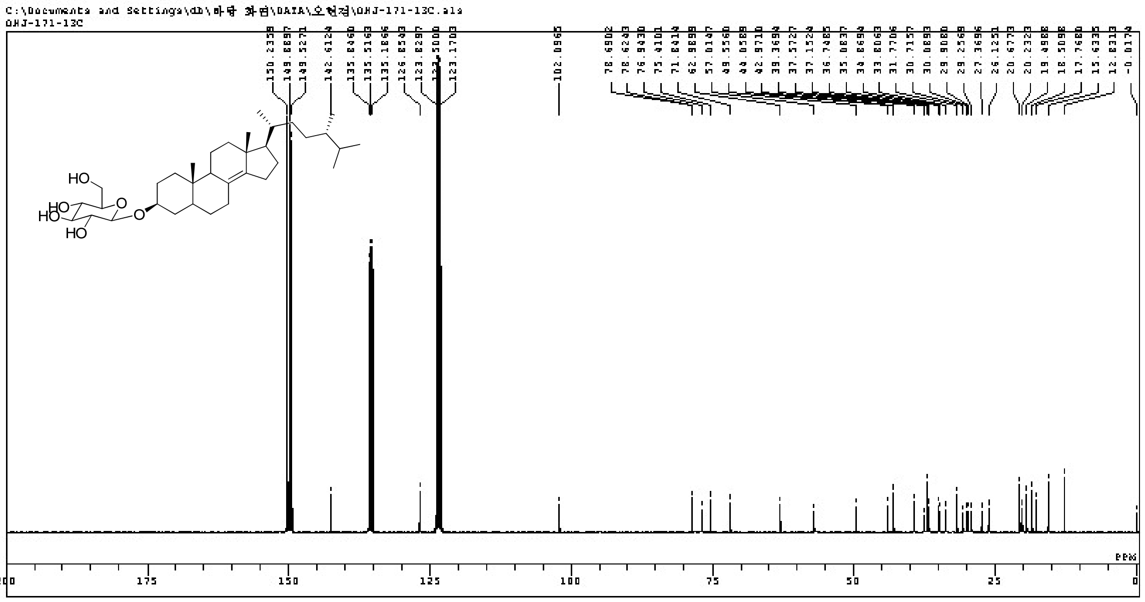

Figure S7. ^{13}C NMR spectrum of compound **3a**

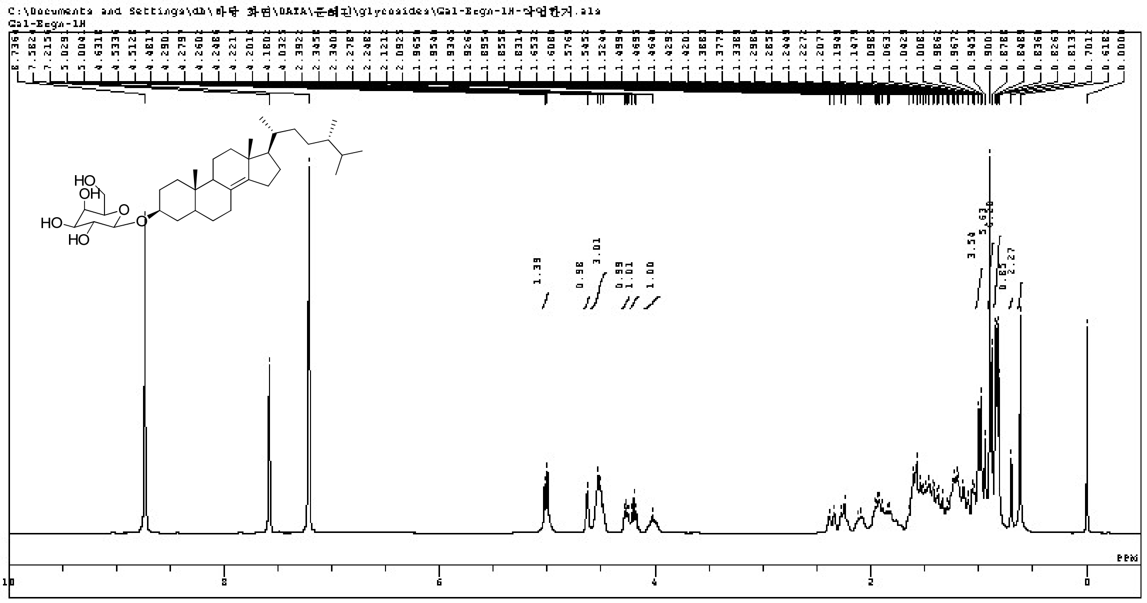

Figure S8. ^{1}H NMR spectrum of compound **3b**

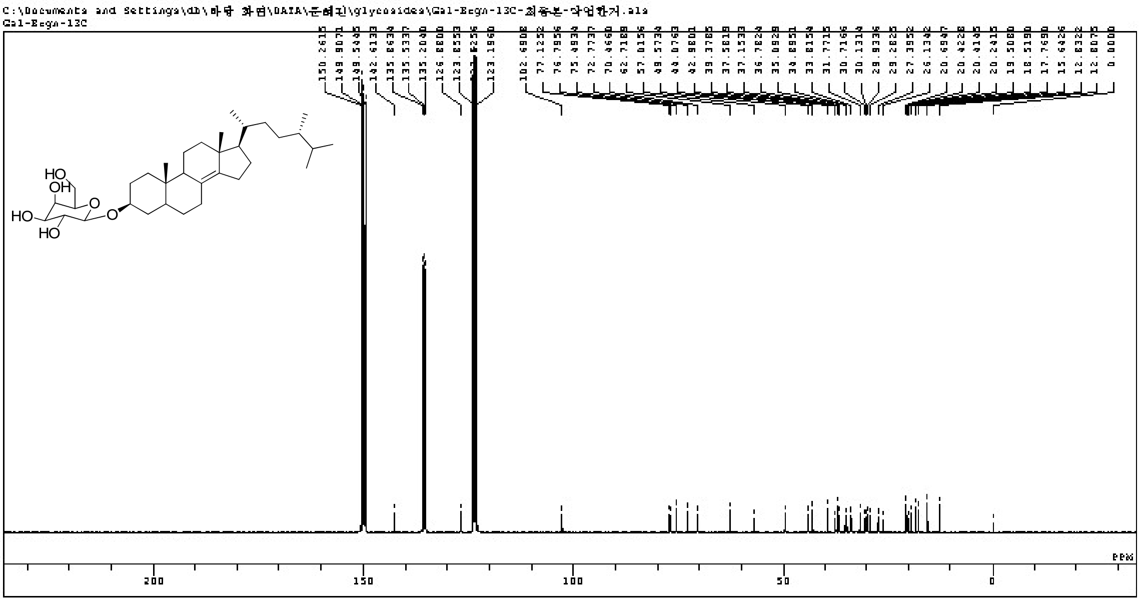

Figure S9. ^{13}C NMR spectrum of compound **3b**

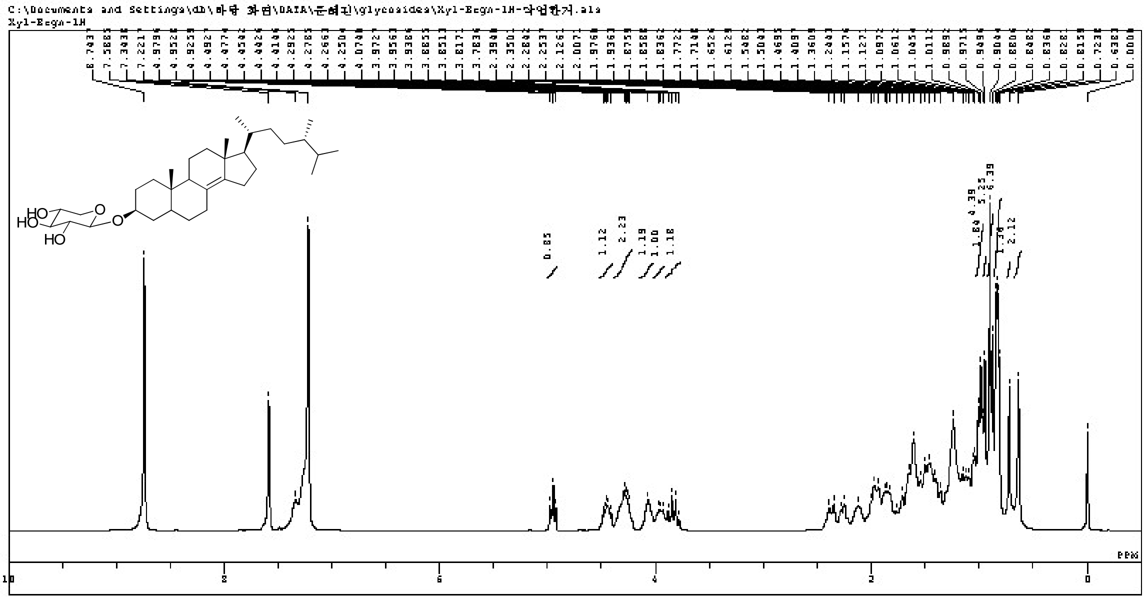

Figure S10. ^{1}H NMR spectrum of compound **3c**

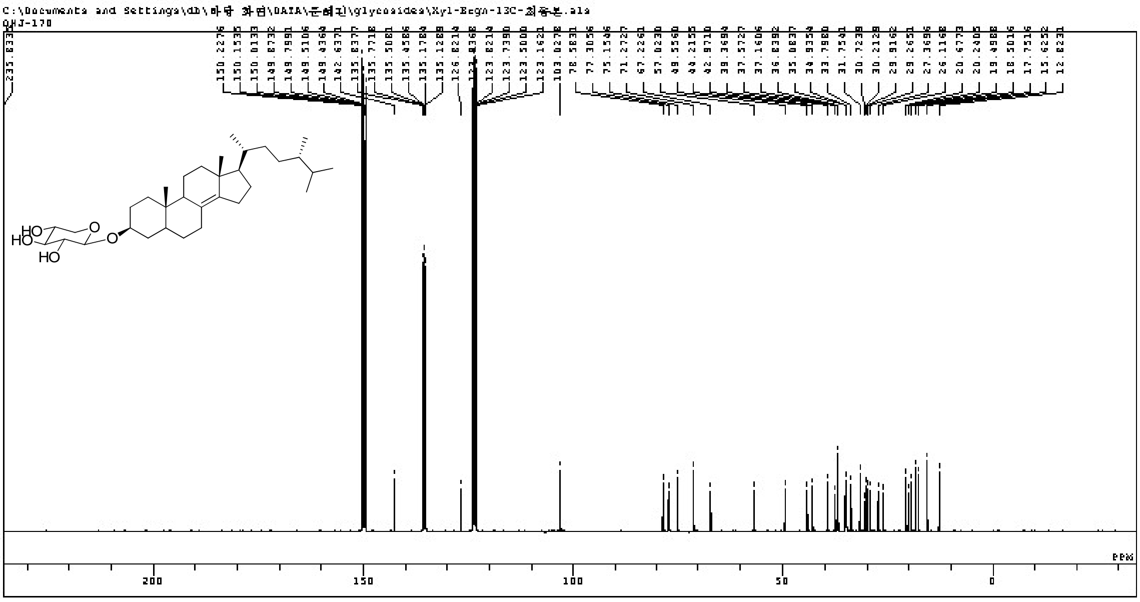

Figure S11. ^{13}C NMR spectrum of compound **3c**
